# Robust thalamic nuclei segmentation method based on local diffusion magnetic resonance properties

**DOI:** 10.1007/s00429-016-1336-4

**Published:** 2016-11-25

**Authors:** Giovanni Battistella, Elena Najdenovska, Philippe Maeder, Naghmeh Ghazaleh, Alessandro Daducci, Jean-Philippe Thiran, Sébastien Jacquemont, Constantin Tuleasca, Marc Levivier, Meritxell Bach Cuadra, Eleonora Fornari

**Affiliations:** 1 Department of Radiology, Lausanne University Hospital (CHUV) and University of Lausanne (UNIL), 1011 Lausanne, Switzerland; 2 Department of Neurology, Icahn School of Medicine at Mount Sinai, One Gustave L Levy Pl, Annenberg Building, Floor 20, Room 81, New York, NY 10029 USA; 3 Centre d’Imagerie BioMédicale (CIBM), Lausanne University Hospital (CHUV) and University of Lausanne (UNIL), 1011 Lausanne, Switzerland; 4 Department of Clinical Neuroscience, Neurosurgery Service and Gamma Knife Center, Lausanne University Hospital (CHUV), 1011 Lausanne, Switzerland; 5 Medical Image Processing Lab (MIPLab), Ecole Polytechnique Fédérale de Lausanne (EPFL), 1015 Lausanne, Switzerland; 6 Signal Processing Laboratory (LTS5), Ecole Polytechnique Fédérale de Lausanne (EPFL), 1015 Lausanne, Switzerland; 7 Department of Pediatrics, University Hospital Center Sainte-Justine, Montreal, H3T 1C5 Canada; 8 Faculty of Biology and Medicine, University of Lausanne (UNIL), Lausanne, Switzerland

**Keywords:** Thalamic nuclei, Segmentation, Orientation distribution function, Spherical harmonics

## Abstract

The thalamus is an essential relay station in the cortical–subcortical connections. It is characterized by a complex anatomical architecture composed of numerous small nuclei, which mediate the involvement of the thalamus in a wide range of neurological functions. We present a novel framework for segmenting the thalamic nuclei, which explores the orientation distribution functions (ODFs) from diffusion magnetic resonance images at 3 T. The differentiation of the complex intra-thalamic microstructure is improved by using the spherical harmonic (SH) representation of the ODFs, which provides full angular characterization of the diffusion process in each voxel. The clustering was performed using the *k*-means algorithm initialized in a data-driven manner. The method was tested on 35 healthy volunteers and our results show a robust, reproducible and accurate segmentation of the thalamus in seven nuclei groups. Six of them closely matched the anatomy and were labeled as anterior, ventral anterior, medio-dorsal, ventral latero-ventral, ventral latero-dorsal and pulvinar, while the seventh cluster included the centro-lateral and the latero-posterior nuclei. Results were evaluated both qualitatively, by comparing the segmented nuclei to the histological atlas of Morel, and quantitatively, by measuring the clusters’ extent and the clusters’ spatial distribution across subjects and hemispheres. We also showed the robustness of our approach across different sequences and scanners, as well as intra-subject reproducibility of the segmented clusters using additional two scan–rescan datasets. We also observed an overlap between the path of the main long-connection tracts passing through the thalamus and the spatial distribution of the nuclei identified with our clustering algorithm. Our approach, based on SH representations of the ODFs, outperforms the one based on angular differences between the principle diffusion directions, which is considered so far as state-of-the-art method. Our findings show an anatomically reliable segmentation of the main groups of thalamic nuclei that could be of potential use in many clinical applications.

**Electronic supplementary material:**

The online version of this article (doi:10.1007/s00429-016-1336-4) contains supplementary material, which is available to authorized users.

## Introduction

The thalamus, which is involved in the regulation of several sensorimotor and cognitive functions, acts as a relay station between cortical and subcortical areas. Many neural signals directed towards the cortex are routed through the thalamus via long ascending fiber tracts, while short fibers connect the thalamus to deep gray matter structures and cerebellum. The thalamus has a complex architecture, made of small cytoarchitectonically subdivided nuclei (Sherman and Guillery 2002), which are connected to each other by intra-thalamic fibers. These nuclei mediate the thalamus’s involvement in a wide range of neurological functions and, therefore, are of key importance in many neurodevelopmental and neurodegenerative disorders.

The automatic segmentation of the thalamic nuclei in vivo using magnetic resonance imaging (MRI) has been limited by the difficulty of obtaining high-resolution images with sufficient contrast and by the lack of appropriate MRI-based features (Gringel et al. 2009; Tourdias et al. 2014). The majority of the published studies for thalamic nuclei segmentation are based on information derived from diffusion-weighted MR imaging (DWI). These approaches use local diffusion properties, such as the full diffusion tensor (Duan et al. 2007; Jonasson et al. 2007; Rittner et al. 2010; Wiegell et al. 2003) and principal diffusion directions (Kumar et al. 2015; Mang et al. 2012; Ye et al. 2013; Ziyan et al. 2006; Ziyan and Westin 2008), global diffusion properties utilizing long-distance projections of each nucleus to the cortex (Behrens et al. 2003; O’Muircheartaigh et al. 2011), or a combination of both local and global diffusion properties (Stough et al. 2014). These approaches are of potential interest, but they present several drawbacks. Some of them use data acquired with a relatively low number of diffusion gradient directions (Jonasson et al. 2007; Kumar et al. 2015; Mang et al. 2012; Wiegell et al. 2003), while others can only identify few nuclei within the thalamus (Stough et al. 2014; Ye et al. 2013). Importantly, most methods require a prior knowledge for the primer initialization and give an outcome that is very sensitive to it (Behrens et al. 2003; O’Muircheartaigh et al. 2011; Stough et al. 2014; Wiegell et al. 2003; Ye et al. 2013; Ziyan et al. 2006; Ziyan and Westin 2008). Overall, robustness and consistency could not be properly evaluated because most of these methods have been tested in only a few subjects (Behrens et al. 2003; Duan et al. 2007; Rittner et al. 2010; Wiegell et al. 2003; Ye et al. 2013; Ziyan et al. 2006; Ziyan and Westin 2008).

Tractography-based approaches (Behrens et al. 2003; O’Muircheartaigh et al. 2011) represent an interesting alternative to the aforementioned local-based ones. They provide functionally reliable clusters (Johansen-Berg, et al. 2004), although these clusters do not necessarily correspond to cytoarchitectonic delineation (Morel et al. 1997). Moreover, they are of limited use if the subject has abnormal white matter status or in the presence of large focal brain lesions, like tumors or vascular lesions. In such cases, fiber reconstruction algorithms can easily fail to identify the connectivity patterns.

The primary objective of this work is to introduce a novel segmentation framework for delineating the thalamic nuclei. The originality of our method is the use of the complete orientation distribution functions (ODFs) rather than a summary statistics, using diffusion MR images at 3 T. The use of spherical harmonics (SH) for the ODFs representation provides full angular characterization of the diffusion process at each voxel.

The framework was tested on 35 healthy volunteers. The diffusion data were acquired using a diffusion-weighted imaging (DWI) sequence widely used in a clinical setting, with the aim of potentially providing a useful tool in everyday clinical practice.

The evaluation of the results was performed both qualitatively, by an experienced neuroradiologist who compared them to a histological atlas, and quantitatively, by measuring clusters’ extent and clusters’ spatial distribution across subjects and hemispheres. We further assessed the reproducibility of our findings using a scan–rescan analysis as well as the robustness of our method across different MR scanners and sequence parameters. At last, we compared our results with the organization of the long connections between each thalamic nucleus and its projections depicted by diffusion MR-based tractography. Our approach could be of potential interest for studying brain anatomy in healthy subjects and for clinical purposes in patients with subcortical white matter lesions or tumors where global thalamo-cortical tractography cannot be performed.

## Materials and methods

The local institutional review board approved the study and all participants gave written informed consent.

### Data

The core of the research project (build-up of the segmentation pipeline, qualitatively and quantitative evaluation of the results) was built using subjects whose demographic characteristics are described in section Dataset 1. We further assessed robustness across different sequences and scanners, and intra-subject reproducibility of the thalamic clusters using two additional datasets (Dataset 2 and Dataset 3).

### Dataset 1

Thirty-seven healthy subjects with no history of neurological illnesses, aged 20–70 years (mean ± std, 42.5 ± 12 years), were recruited. The exclusion criterion was the presence of white matter alterations visible on fluid-attenuated inversion recovery (FLAIR) images examined by an experienced neuroradiologist. Two subjects were excluded because of technical problems during MRI acquisition leading to a final dataset of 35 control subjects. All subjects were scanned in a 3-T Siemens Trio scanner (Siemens AG, Erlangen, Germany) using a 32-channel head coil. The protocol included a sagittal T1-weighted gradient-echo sequence (MPRAGE), 160 contiguous slices, 1-mm isotropic voxel, repetition time (TR) 2300 ms, echo time (TE) 2.98 ms, field of view 256 mm as a basis for segmentation. FLAIR contrast images were acquired with a voxel size of 0.9 × 0.9 × 2.5 mm^3^, flip angle 150°, TR 9500 ms, TE 84 ms, 32 axial slices. Diffusion-weighted images were acquired using a spin-echo echo-planar imaging sequence (64 gradient directions, *b* value 1000 s/mm^2^, voxel size 2 × 2 × 2.5 mm^3^, 52 axial slices, TR 6700 ms, TE 89 ms, field of view 192 × 192 mm) plus 1 volume without diffusion weighting (*b* value 0 s/mm^2^, i.e. *b*_0_) at the beginning of the sequence as anatomic reference for motion and eddy current correction.

### Dataset 2

Six healthy males (30.2 ± 6.2 years) were imaged with a 3-T Prisma Siemens scanner (Siemens AG, Erlangen, Germany). For all of them, an identical diffusion sequence was acquired twice the same day using the following parameters: TR/TE = 7800/78 ms, flip angle = 90°, 60 gradient directions with *b* value = 2000 s/mm^2^, voxel size of 2 x 2 x 2 mm^3^, 60 axial slices and 10 volumes without diffusion weighting. Additional MPRAGE was obtained with TR/TE = 2300/2 ms, flip angle = 9°, voxel size of 1 × 1 × 1.2 mm^3^, 160 axial slices.

### Dataset 3

The third dataset was composed of two elderly essential-tremor patients (2 males, 86 years of age) treated with Gamma Knife thalamotomy. The images were acquired at two different time points: the day before the treatment and 6 months after using a 3-T Prisma Siemens scanner. The parameters for the diffusion sequence were similar to those used for Data 1: TR/TE = 7100/84 ms, flip angle = 90°, 64 gradient directions with *b* value = 1000 s/mm^2^, voxel size of 2.2 x 2.2 x 2.2 mm^3^, 62 axial slices and 10 volumes without diffusion weighting. The corresponding MPRAGEs on both dates were obtained with TR/TE = 2300/2 ms, flip angle = 9°, voxel size of 1 × 1 × 1.2 mm^3^, 160 axial slices. Both patients underwent Gamma Knife surgery on their left thalamus, and consequently, we performed analyses only on their right thalamus.

### Pre-processing

Diffusion-weighted images were first filtered using an isotropic Gaussian kernel (*σ* = 0.8 mm^3^) and then analyzed with FSL (http://www.fmrib.ox.ac.uk/fsl/index.html). The pre-processing of the diffusion dataset (64 gradient directions) involved motion and eddy current correction. In this step, each diffusion-weighted image was registered to the *b*_0_ image (no diffusion encoding) using a 12-parameter affine transformation. This transformation accounts for motion between scans and residual eddy current distortions present in the diffusion-weighted images. The diffusion tensor was then estimated (Mori and Zhang 2006) and the three eigenvalues of the tensor were used to compute the fractional anisotropy (FA) map for each subject on a voxel-by-voxel basis (Pierpaoli and Basser 1996). This scalar measure of white matter fiber integrity was used to refine the segmentation of the thalamus (see section “Thalamus extraction” for details).

In addition, the T1-weighted image was automatically segmented in the subject’s native space in gray matter (GM), white matter (WM), and cerebrospinal fluid (CSF) using the unified segmentation approach (Ashburner and Friston 2005) implemented in SPM8 (Wellcome Trust Centre for Neuroimaging: http://www.fil.ion.ucl.ac.uk/spm/) running under Matlab 7.11 (MathWorks Inc, Sherborn, MA, USA). The T1-weighted image was registered to the diffusion space using a rigid-body transformation with 6 degrees of freedom and Mattes Mutual Information as cost function (Johnson et al. 2007). The same transformation was then applied to the CSF probability map. The CSF image, together with the FA image, were used to increase the accuracy of the automatic thalamus extraction as described in the following paragraph.

### Thalamus extraction

**Fig. 1:**
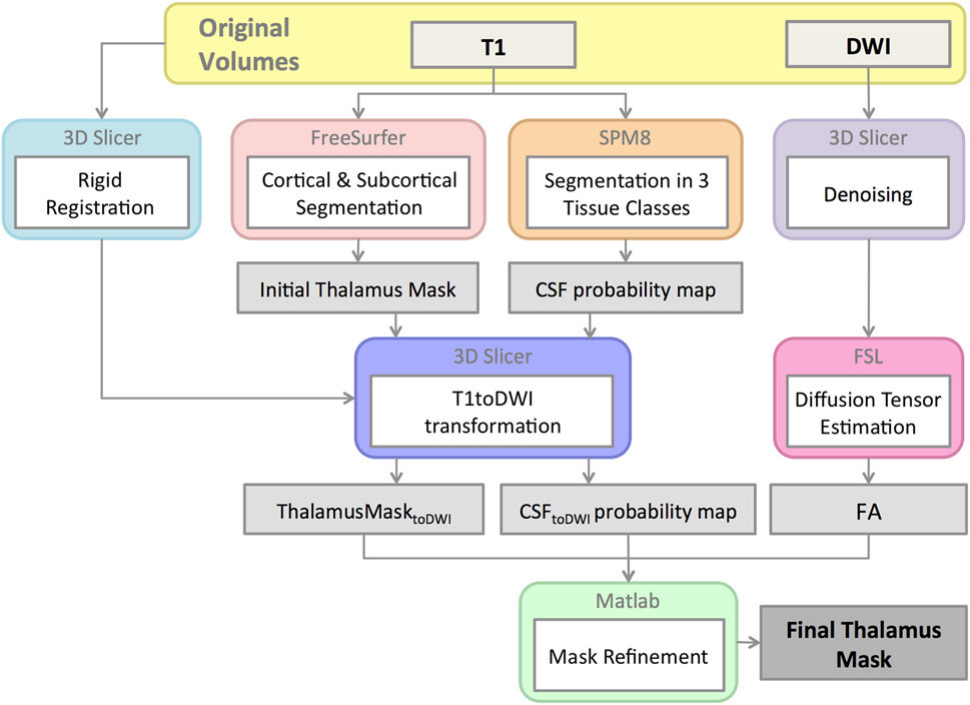
Outline of the main pre-processing steps for accurate thalamus extraction

The processing steps to obtain an accurate mask of the whole thalamus are summarized in Fig. 1. First, we performed cortical and subcortical parcellation of the T1-weighted images with the FreeSurfer software (http://surfer.nmr.mgh.harvard.edu). The subcortical parcellation includes the pre-processing of the MRI data (bias correction, intensity normalization) and the subcortical labeling of the tissues classes (Fischl et al. 2002, 2004). Second, the labels corresponding to the right and the left thalamus were identified, converted to binary masks, and registered to the diffusion space by applying the previously estimated transform (details in “Pre-processing” section). Third, the registered binary masks of the thalamus were then refined using the CSF and FA maps. To exclude partial volume contaminations, we only considered voxels with CSF probability value lower than 0.05. In addition, to avoid partial volume of the internal capsule in the proximity of the thalamus, voxels within a 2-mm distance from the border of the mask with FA values greater than 0.55 were also excluded. All these steps were performed in each subject’s diffusion space.

### Reconstruction of the orientation distribution functions (ODFs)

The orientation distribution functions (ODFs, Eq. 1) were computed using q-ball imaging in constant solid angle (Aganj et al. 2010) using the *Qboot* tool available in FSL:

1 $${\text{ODF}}_{\text{CSA}} \left( \varvec{u} \right) \propto {\text{FRT}}\left\{ {\Delta_{b}^{2} { \ln }\left( { - \ln \frac{{S\left( \varvec{u} \right)}}{S\left( 0 \right)}} \right)} \right\}$$

where FRT is the Funk Radom transform, and $\Delta_{b}^{2}$ the Laplace–Beltrami operator. The diffusion signal *S* was modeled by means of the real and symmetric spherical harmonic (SH) basis as in Descoteaux et al. 2007:

2 $$\ln \left( { - { \ln }\frac{{S\left( \varvec{u} \right)}}{S\left( 0 \right)}} \right) = \mathop \sum \limits_{j = 1}^{{\left( {l + 1} \right)\left( {l + 2} \right)/2}} c_{j} Y_{j } \left( \varvec{u} \right) + e_{\text{bstr}}$$

with *c*_*j*_ the coefficient of the *j*th SH basis function $Y_{j }$, *l* the maximum SH basis order, and *e*_bstr_ the Bootstrapped residual.

For each subject, the *Qboot* algorithm was applied by setting the maximum number of ODF peaks to be detected to 2 using 50 samples for residual bootstrapping (Whitcher et al. 2008), as in the default settings of the *Qboot* command in FSL. The maximum SH basis order was instead set to 6 (*l* = *6*). Results of the *Qboot* bootstrapping were samples of ODF shapes for each voxel, and the mean coefficients of each voxel served as inputs to the clustering algorithm.

**Fig. 2:**
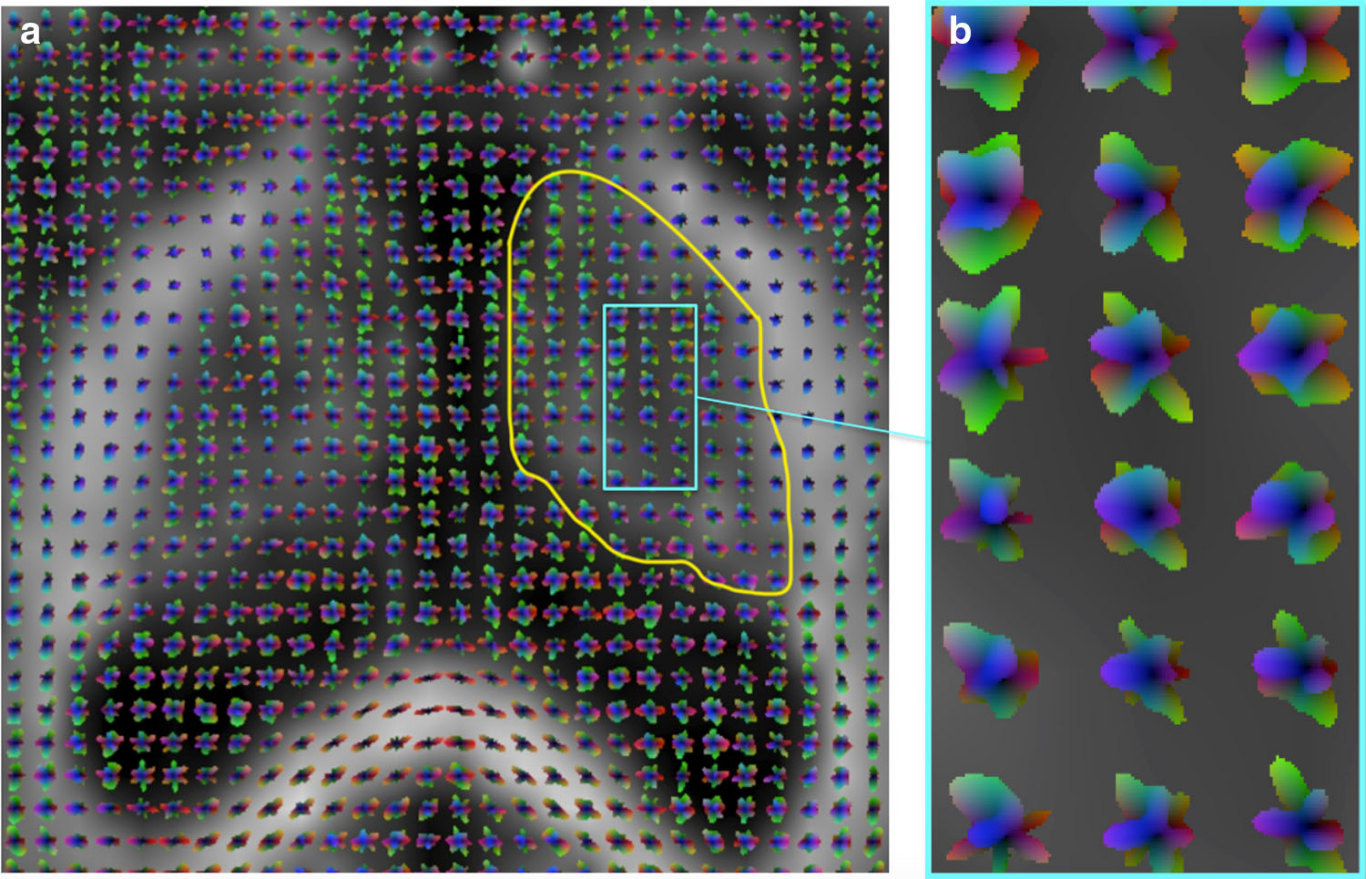
Visualization of the ODFs in a slice of the thalamus. The *yellow contour* in **a** delineates the thalamus, while **b** provides a close-up view of the ODFs shapes inside the thalamic area identified by the *light-blue box*

The SH basis allows a full angular characterization of the ODFs (Fig. 2) by means of real-SH vectors. Therefore, it was possible to assess similarities of diffusion properties across ODFs using simple distance metrics (Wassermann et al. 2008).

### Clustering of the thalamic nuclei

**Fig. 3:**
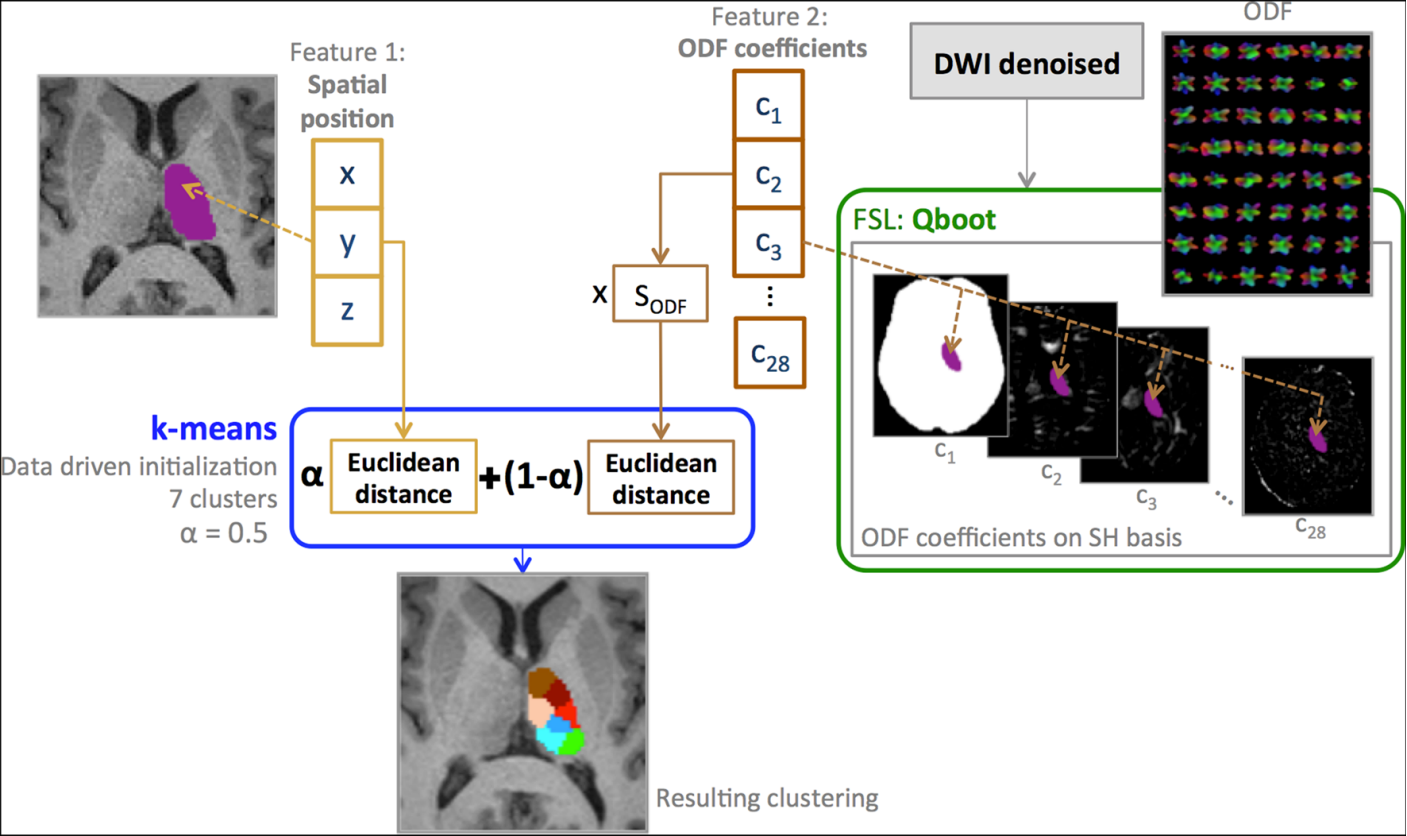
Schematic overview of the clustering framework. Segmentation of the seven thalamic nuclei has been performed using a *k*-means clustering algorithm with two equally weighted features: the spatial position of the voxels inside the thalamus (*x, y, z*) and the mean ODF coefficients (*C*_*i*_ *, i* ∈ [1, 28]) expressed in the SH basis of maximum order 6. *k*-means is initialized in a data-driven fashion

Clustering was performed using a modified unsupervised *k*-means algorithm. A schematic overview of our method is shown in Fig. 3. Inputs were mean SH coefficients and voxel position. The number of clusters to be segmented was set to seven based on a preliminary analysis that used a lower number of subjects aimed at determining the maximum number of clusters that would provide a robust segmentation pattern across subjects. Additionally, previous studies subdivided the thalamus in seven nuclei (Behrens et al. 2003; O’Muircheartaigh et al. 2011).

The decision metric for the final clustering was a combination of the Euclidean distance of the voxels position and of the Euclidean distance calculated from the SH coefficients (Eq. 3):

3 $$||{\text{ODF}} - {\text{ODF}}^{\prime }|| = \sqrt {\mathop \sum \limits_{j = 1}^{R} \left( {c_{j} - c{\prime }_{j} } \right)^{2} }$$

To avoid any bias in the *k*-means clustering, we applied a scaling factor (*S*_ODF_) to the SH coefficients to scale the ODF distances inside the interval of the spatial-distance values. The scaling factor *S*_ODF_ = 55 was first empirically estimated on a small group of subjects and then applied to the remaining dataset. The contribution of the two features to the final clustering was equal, i.e. the weight *α* was set to 0.5.

To avoid dependency of the results on the initialization method, we first ran 5000 randomly initialized *k*-means, using only the position as the input feature, and then used the average centroid over the 5000 results as the initial setting for the clustering algorithm.

### Evaluation of the results

To assess the robustness of the outcome, we studied the average spatial distribution of the resulting clustering. Clustering results were all registered to the Montreal Neurological Institute (MNI) space using a combination of rigid, affine, and B-spline transformations (with 5 as maximum allowed displacement of the control grid along each axis) implemented in 3D Slicer (http://www.slicer.org). We then created a weighted average map in which each voxel was defined with the label value represented by the majority of subjects in that voxel (we will further refer to it as weighted mean map by majority voting or just mean segmentation map).

The assessment of the thalamic nuclei clustering is extremely challenging due to the absence of a *gold standard*, and this limitation is shared by all previously proposed techniques. Moreover, none of the methods in the literature evaluated reproducibility across different time points or different diffusion sequences. To this end, four different approaches for evaluating the anatomical consistency of our results were used.

#### (a) Qualitative evaluation

An experienced neuroradiologist (PM) visually assessed the quality of the segmentation results and further compared them to Morel histological atlas (Morel et al. 1997).

#### (b) Quantitative evaluation

i. *Symmetry between the left and the right thalamus*. To test the symmetry between results of the left and right thalamus, we statistically compared the volume and the spatial distribution of the centroids of each segmented cluster between the left and the right hemisphere using a non-parametric Wilcoxon signed-rank test. All analyses were performed on the subjects’ diffusion space. For each hemisphere, each cluster volume was normalized by the size of the thalamus to take into account the inter- and the intra-individual size variability. The distribution of the centroids was calculated using a distance map representing the relative position of the centroids’ coordinates to the closest contour of the thalamus mask.

ii. *Intra-subject reproducibility*. We assessed intra-subject variability using scan–rescan data from Dataset 2 and Dataset 3. For each subject, we performed the clustering on both time points scans separately. The resulting clusters obtained from each dataset were brought to the same image space by applying a rigid 6-parameter transformation, which was estimated with 3D Slicer (Johnson et al. 2007). Finally, clusters of scan–rescan time points were quantitatively compared using:

– Dice’s coefficient for assessing the overlap

– Euclidean distance between the centroids

– Modified Hausdorff distance for evaluating the similarity between the cluster contours. The modified distance has been shown to be more robust to outliers than the traditional Hausdorff distance (Dubuisson and Jain 1994).

#### (c) Comparison with thalamic long connections

The behavior of our algorithm, which uses local information derived from DWI, was compared to the organization of the long fibers connections between the thalamus and its afferent and efferent projections. We used probabilistic tractography (computed with probtrackx from FSL package) to highlight those pathways. Based on anatomy (Jones 1985), we first identified, for each group of thalamic nuclei, the regions characterizing its afferent and efferent connections. The mask of the whole left thalamus was maintained as a constant seed region in the tractography, while target masks were chosen according to the regions representing the two endpoints of each specific pathway of interest.

The results of the tractography showed the portion of the thalamus whose fibers were connected to the target masks. We then compared the location of those subparts of the thalamus with our clustering results. For each cluster, we defined the frequency of success (FS) as the percentage of subjects in which the tract of interest overlapped the expected cluster.

For each cluster in each subject, probabilistic streamlines were computed using the modified Euler integration (Cordova and Pearson 1988), by drawing 7000 individual samples using a value of 0.5 mm for step length and 0.2 for curvature threshold. To reduce potential bias from spurious tracts, we have excluded voxels having probabilistic streamlines value below 5% of the maximum. All the streamlines between the respective that survive this threshold were considered as part of the tracts of interest and included in a mask.

#### (d) Comparison with state-of-art methods based on local diffusion properties

Up to date, the angular difference (AD) between the principle directions of the diffusion tensor was considered as the most reliable local feature for thalamic nuclei parcellation (Ziyan et al. 2006). To assess our contribution and the advantage of using SH representations of the ODFs over existing techniques, we compared the results of our pipeline with those obtained using AD as feature. First, we computed the diffusion tensor at each voxel with FSL diffusion toolbox, and then, instead of using the Euclidean distance between the ODF coefficients inside the clustering framework, we calculated the angular difference between the main eigenvector of the diffusion tensor (Kumar et al. 2015; Mang et al. 2012; Ziyan et al. 2006). In order to have both distances in the same range of values within the *k*-means algorithm, we scaled AD after computing it by a factor of 6, which was empirically determined. Comparison between ODF and AD features is done at one time point (using Morel’s atlas for validation in two different axial slices) as well as with scan–rescan setting.

## Results

**Fig. 4:**
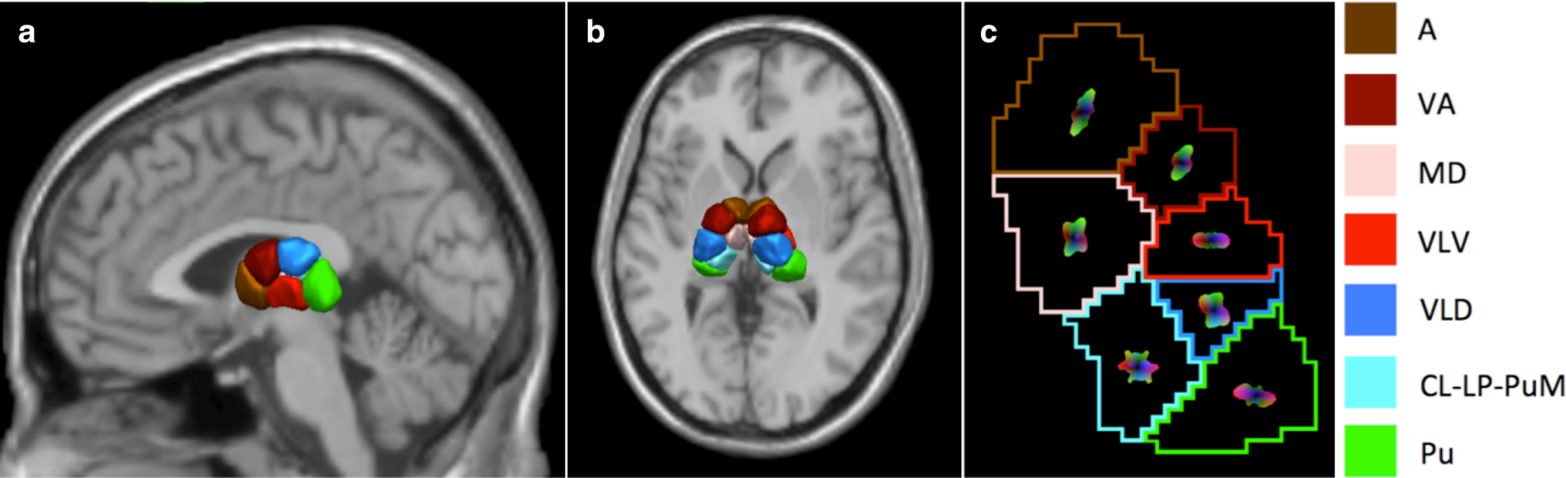
Rendering of the weighted mean clustering map by majority voting. The map is superposed on a T1-weighted image in the Montreal Neurological Institute (MNI) space in sagittal (**a**) and transversal (**b**) views. Panel **c** represents the mean ODF characteristic for each cluster. Each averaged ODFs were reconstructed on a representative subject and superposed on the weighted mean clustering map. Thalamic nuclei are color-coded as follows: *brown* for the anterior group (A), *maroon* for the ventral anterior group (VA), *light pink* for the medio-dorsal group (MD), *red* for the ventral latero-ventral group (VLV), *blue* for the ventral latero-dorsal group (VLD), *green* for the pulvinar (Pu), and *cyan* for the cluster representing the central lateral nucleus, the lateral posterior and a portion of the medial part of the pulvinar (CL–LP–PuM)

**Fig. 5:**
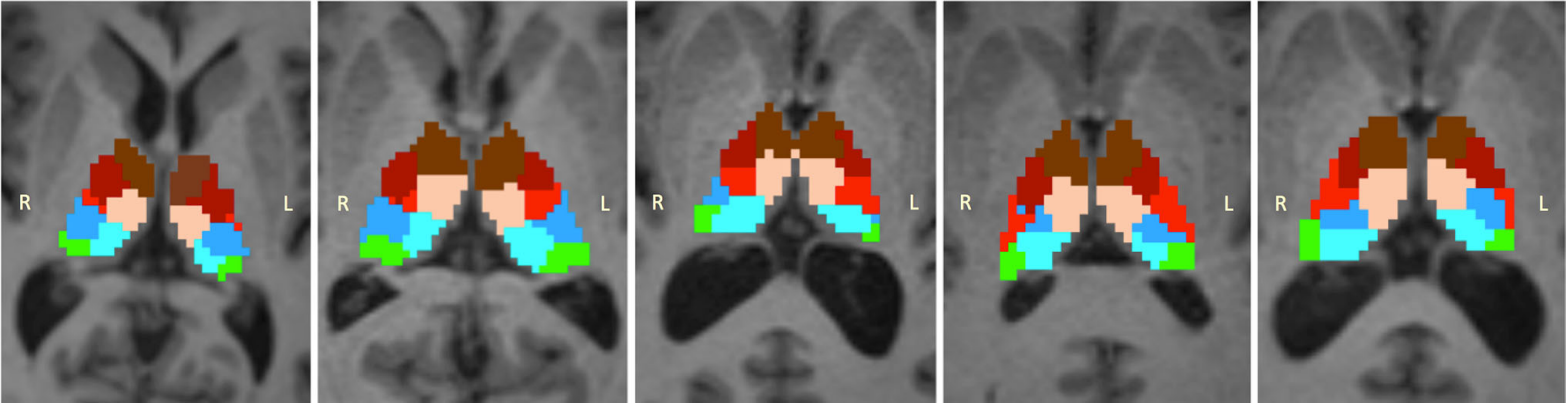
Individual results of the thalamic nuclei segmentation. Spatial distribution of the segmented nuclei are shown in axial view for five different cases and superposed on each subject’s MPRAGE image

The thalamic nuclei clustering in Dataset 1 was highly reproducible and characterized by a robust pattern of spatial distribution. Only one subject out of the 35 deviated from this pattern. In fact, he presented an intensity spike in ODF coefficients’ values as an artifact of the reconstruction that anomalously biased the clustering. Therefore, this subject was removed from further analysis. The mean segmentation map that represents the spatial distribution pattern is shown in Fig. 4, while Fig. 5 gives an example of five individual results.

*(a) Qualitative evaluation*  

**Fig. 6:**
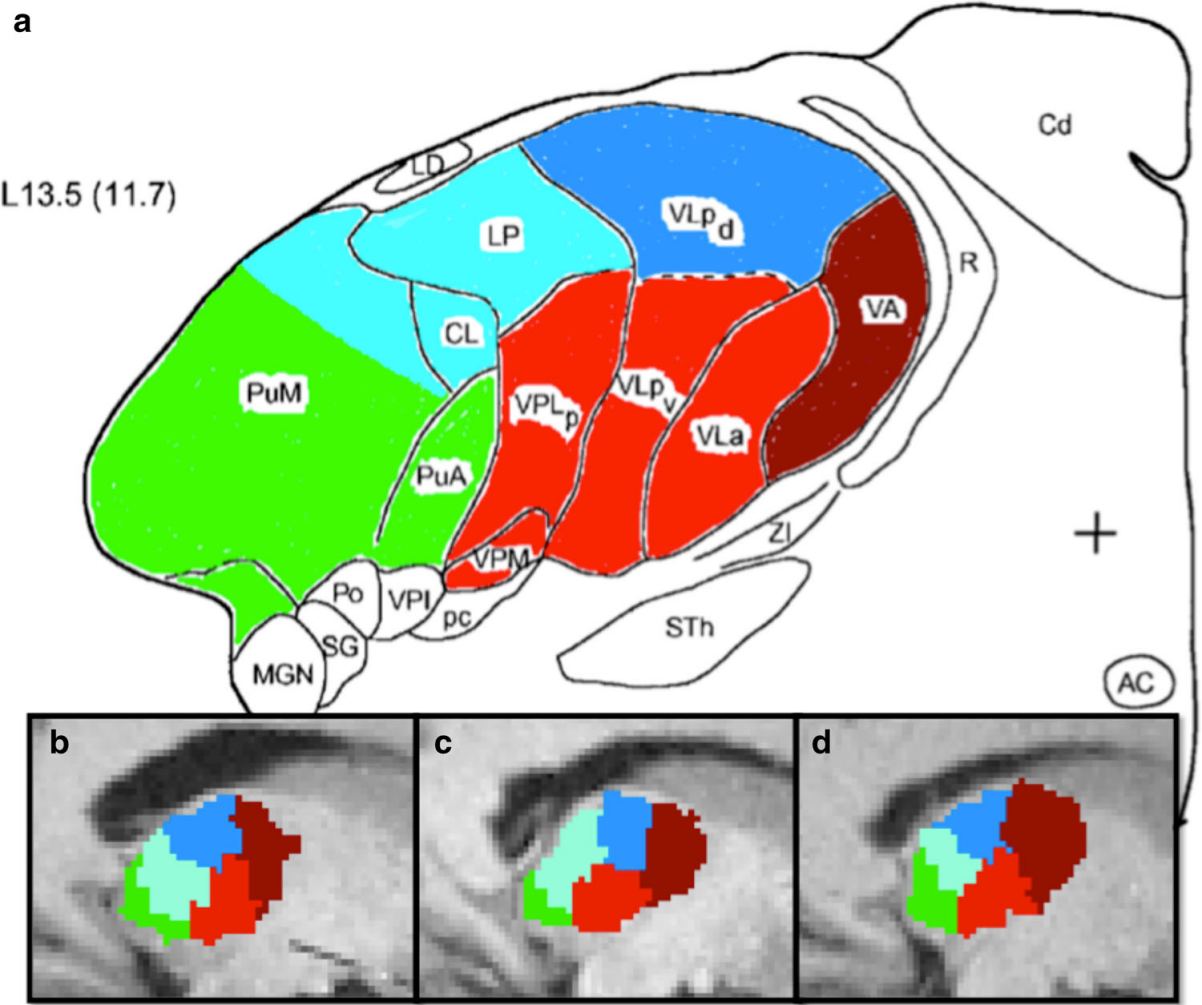
Comparison of the results of our clustering algorithm with the Morel’s histological atlas. **a** shows a sagittal view of the Morel atlas. **b**–**d** show instead the spatial distribution of the thalamic nuclei segmented with our framework in the same sagittal slice for three different cases in the Talairach space. *Each color* gives the anatomical correspondence of each group of nuclei

**Fig. 7:**
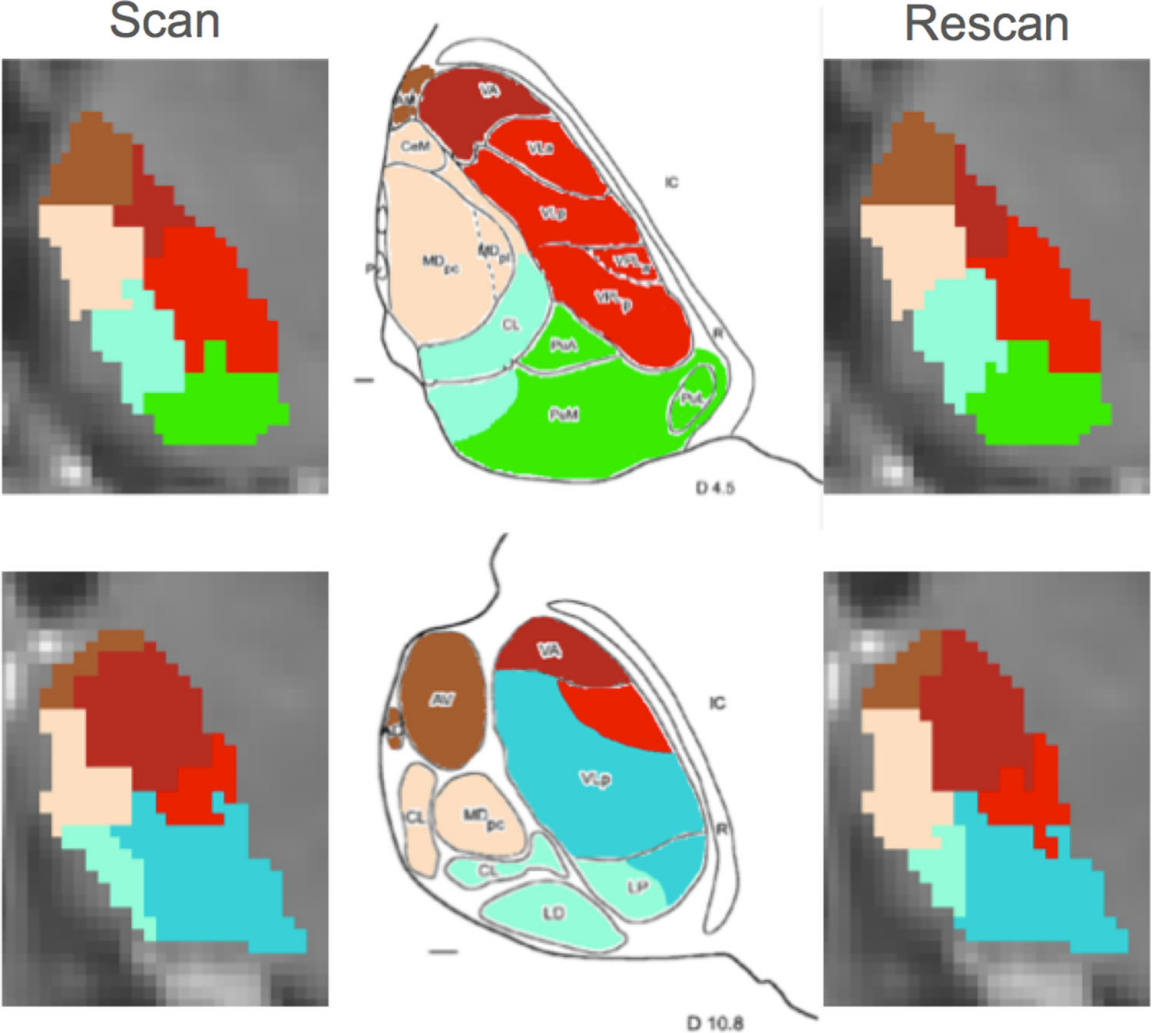
Resulting clustering from the scan–rescan analysis compared with two different axial slices from the Morel’s atlas (*D* 4.5 and *D* 10.8 *top* and *bottom row*, respectively)

For each subject, the expert evaluated the spatial distribution and extent of the clusters segmented with our algorithm, and while comparing them to Morel’s atlas, he labeled each cluster by its anatomical correspondence (see Figs. 4, 6, 7). Six out of seven clusters could be uniquely identified as a known anatomical nucleus or group of nuclei, and we, therefore, assigned the name of the dominant nucleus to each of them in each respective group. The seventh cluster instead, was characterized by two predominant nuclei, the central lateral (CL) and the lateral posterior (LP), as well as by a portion of the anterior part of the medial pulvinar (PuM). The anatomical partitions derived from our clustering were labeled as follows: anterior group (A), ventral anterior group (VA), medio-dorsal group (MD), ventral latero-ventral group (VLV), ventral latero-dorsal group (VLD), pulvinar (Pu) and CL–LP–PuM group (see Figs. 4, 6, 7).

Based on the qualitative comparison with the histological atlas, one subject did not pass the expert evaluation, since the spatial distribution of the segmented clusters deviated from the one of the other 33 cases. We assume that such an outcome is due to large neuroanatomical variation, but since it represented an outlier, we decided to exclude this subject from further evaluation analyses.

*(b) Quantitative evaluation*

i. *Symmetry between the left and the right thalamus*. We observed an important symmetry between the results on the left and on the right thalamus across all subjects, which was confirmed by our statistical analysis. As shown in Tables 1 and 2, respectively, neither the normalized volumes nor the centroids distribution of the corresponding cluster over hemispheres were significantly different.

**Table 1:** Statistical comparison of the normalized volumes of the thalamic nuclei across hemispheres

Volume							
Wilcoxon signed-rank test	Pu	A	MD	VLD	CL–LP–PuM	VA	VLV
*p* value	0.77	0.55	0.5	0.28	0.14	0.63	0.25
Median values (mm)							
Left	0.1319	0.1599	0.1571	0.1239	0.1248	0.1540	0.1371
Right	0.1331	0.1618	0.1606	0.1193	0.1317	0.1513	0.1326

**Table 2:** Statistical comparison of the centroids distribution of the thalamic nuclei across hemispheres

Centroids’ border distance							
Wilcoxon signed-rank test	Pu	A	MD	VLD	CL–LP–PuM	VA	VLV
*p* value	0.75	0.4	0.36	0.49	0.39	0.79	0.24
Median values (mm)							
Left	2	2.2361	2.1180	2	2	2.2361	2
Right	2	2.2361	2.2361	2.2361	2.2361	2.2361	2

ii. *Intra-subject reproducibility*. The resulting clustering from Dataset 2 and Dataset 3 presented the same segmentation pattern as observed for the 33 subjects in Dataset 1. Similarly, the same pattern was observed in the scan–rescan analysis in both datasets. In particular, for all the 14 inspected thalami, the average Dice’s coefficient value per cluster was always higher than 0.8, while centroid’s and Hausdorff distance were lower than the original spatial resolution of the diffusion images used. Table 3 gives a summary of these results, while Fig. 7 and Figs. SM1 and SM2 in the supplementary material show a visual illustration of them, together with additional comparisons with Morel’s atlas.

**Table 3:** Quantitative measures of similarity between the scan–rescan clusters

Measure	Dice coefficients		Centroids’ distance (mm)		Hausdorff distance (mm)	
Cluster	Mean	Variance	Mean	Variance	Mean	Variance
Pu	0.93	0.0007	0.56	0.08	0.17	0.0025
A	0.90	0.0024	0.86	0.34	0.24	0.0036
MD	0.84	0.0080	1.36	1.08	0.28	0.0055
VLD	0.87	0.0018	0.98	0.26	0.27	0.0043
CL–LP–PuM	0.83	0.0101	1.41	1.24	0.28	0.0053
VA	0.89	0.0016	0.79	0.28	0.26	0.0038
VLV	0.89	0.0031	0.66	0.26	0.22	0.0019

*(c) Comparison with thalamic long connections*

According to the anatomy (Jones 1985), we reconstructed six specific pathways, one for each nucleus characterized by a unique anatomical distribution, i.e. A, VA, MD, VLD, VLV, and Pu. The respective pairs of target masks that define the specific pathway for each cluster are summarized in Table 3.

This approach included all clusters except CL–LP–PuM since it is composed by more than one dominant nucleus; thus, its specific pathway could not be uniquely identified.

The estimated average FS for all clusters was 92.4% with minimum value of 81.8% for the VA cluster and maximum of 100% for the pulvinar. More details about FS values for each cluster, respectively, are given in Table 4.

**Table 4:** Summary of the pairs of target masks chosen for the reconstruction of the pathways characteristic of each group of nuclei

Cluster	Target 1	Target 2	FS (%)
A	Anterior cingulate cortex	Fornix	97
VA	Premotor cortex (Broadman area 6)	Substantia nigra	81.8
MD	Middle frontal sulcus	Amygdala	90.9
VLD	Posterior singular cortex	Fornix	87.9
VLV	Precentral gyrus	Red nucleus (left) and superior cerebellar peduncle (right)	97
Pu	Inferior angular gyrus	Calcarine sulcus	100

The frequency of success (FS) was defined as the percentage of subjects for which there was an overlap between the cluster and the thalamic part of the corresponding tract

**Fig. 8:**
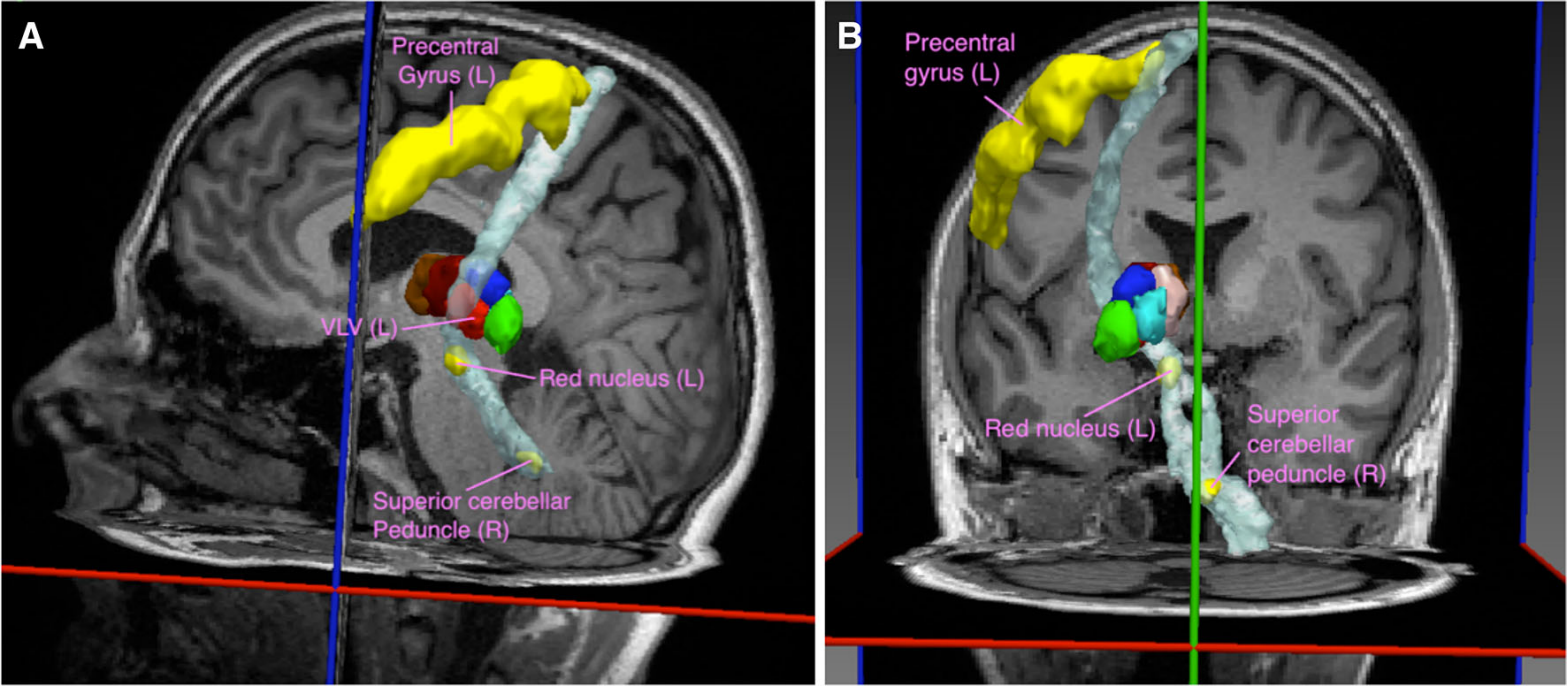
Reconstruction of thalamic long connections. Sagittal (**a**) and coronal (**b**) 3D views of the motor fiber tracts passing through the cluster VLV (in *red*). Probabilistic tracts (in *white*) were reconstructed using the whole thalamus mask and the following seed regions (in *yellow*): left precentral gyrus, left* red* nucleus, and right superior cerebellar peduncle

An illustration of the motor tract passing through the VLV cluster is given in Fig. 8. Examples of the reconstruction of two other tracts are shown in Fig. SM3 of the supplementary material.

*(d) Comparison with state-of-art methods based on local diffusion properties*

Unlike the results given by our ODF-based approach, the AD-based segmentation clustered nuclei whose spatial distribution could not be uniquely assigned to a specific anatomical group, according to the Morel’s atlas (see Fig. 7; Figs. SM1 and SM2 from the supplementary material). We further noticed that the clusters distribution obtained from Dataset 2, characterized by a diffusion acquisition at high *b* value, had less smooth boundaries, noisy contours, and isolated voxels.

**Fig. 9:**
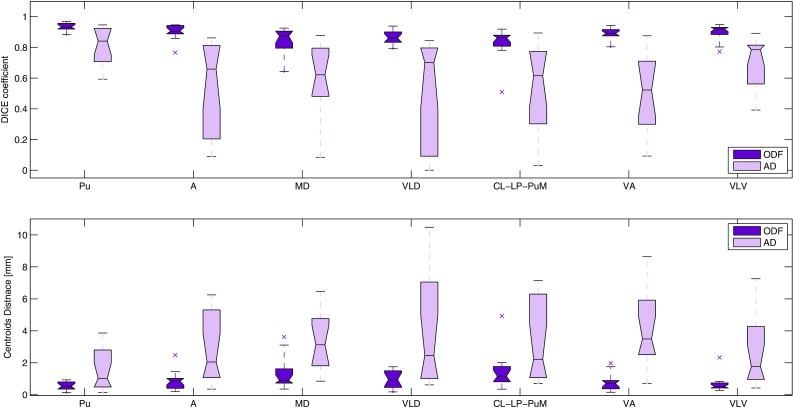
Quantitative measures of overlap between the corresponding clusters in scan–rescan analysis: ODF- versus AD-based segmentation

In the scan–rescan framework, we observed lower intra-subject reproducibility of the AD-based segmentation compared to the ODF-based one. These observations were then confirmed with the quantitative measures showed in Fig. 9. More precisely, the average Dice coefficients per cluster from the AD-based segmentation were between 0.5 and 0.8, while the average distance between the corresponding centroids reached 4 mm.

## Discussion

We presented a novel segmentation framework based on local diffusion properties and spatial features for thalamic nuclei clustering in diffusion MRI. Unlike most of the existing methods, which are limited by the low angular resolution of DWI (Duan et al. 2007; Jonasson et al. 2007; Mang et al. 2012; Rittner et al. 2010; Wiegell et al. 2003; Ye et al. 2013; Ziyan et al. 2006; Ziyan and Westin 2008), ours provides a robust and accurate diffusion-based segmentation by the inclusion of the orientation distribution functions (ODFs) from MR images at 3 T. Our major contribution is the use of spherical harmonics for the ODFs representation that provide full angular characterization of the diffusion processes in each voxel, and, therefore, a better differentiation of the complex intra-thalamic microstructure (Jones 1985). We proved the robustness of our approach across sequences, scanners and acquisitions at different time points. We further demonstrated its outperformance compared to AD-based clustering.

The segmentation was performed using the *k*-means algorithm. Unlike the state-of-the-art methods published so far, the initialization made in a data-driven fashion (primer centroids extracted from 5000 initial randomly-initialized *k*-means runs) adds another strong point to our framework since it is a user-independent procedure. Moreover, such initialization might be a contributing factor to the high reproducibility of the final clustering results.

We segmented the thalamus in seven independent groups of nuclei with a success rate of 97.1% of the tested 35 cases. Six clusters are characterized with unique anatomical distribution, while the importance of the seventh cluster, the CL–LP–PuM, comes from the nuclei grouped within. More precisely, CL is part of the intralaminar nuclei, which are characterized by various connections to frontal and parietal cortices, and potentially involved in arousal mechanisms (Saalmann 2014), while LP together with the pulvinar take part in attention processes to visual stimuli (Swenson 2006). The choice of the number of nuclei was based on a preliminary analysis aiming at identifying the number of clusters that provide a robust segmentation pattern and was further supported by the existing approaches used in the literature. Thalamic nuclei segmentation using thalamo-cortical projections (Behrens et al. 2003) used seven cortical targets to draw probability distribution of connections from voxels within the thalamus to those regions that have been shown to correspond to known connection areas of major thalamic nuclear groups. On the other hand, the myelo- (Magnotta et al. 2000) and cytoarchitectonical (Morel et al. 1997) atlases, which, instead, provide histological information about the structural organization of the thalamus, give a more complete and detailed picture of the thalamic nuclei even though they are built on very limited number of specimens, and therefore, they do not account for any anatomical variability. Nevertheless, as in the Morel atlas (Morel et al. 1997), all nuclei can be spatially grouped into seven main groups. In addition, the number of clusters used in our study seemed to be a good trade-off between spatial resolution of the ordinary DWI acquisition and anatomical accuracy of the clustering. For instance, a recent work (Kumar et al. 2015) attempted to segment the thalamus in 21 different clusters but only five of them appeared to be consistent across subjects. Collectively, these considerations suggest that the robustness of the segmentation method is preserved solely for a small number of clusters when utilizing classical diffusion sequences.

The developed framework was tested in a main dataset of 35 healthy volunteers, which is a relatively large dataset compared to the data used for testing the majority of the existing methods. Validation also remains a challenge for all previously published methods. By employing four different evaluation approaches to assess the results (qualitative comparison of the segmented clusters to the cytoarchitectonic atlas, quantitative analysis of cluster spatial extent and volume across hemispheres, as well as intra-subject reproducibility and correspondence of the thalamic clusters distribution to thalamic long connections), we ensured thorough validation of our algorithm.

A high degree of symmetry of nuclei volume and spatial distribution is in accordance with previous studies using fiber-tracking connectivity-based clustering (Behrens et al. 2003), functional information derived from resting-state BOLD signal (Hale et al. 2015; Kim et al. 2013; Zhang et al. 2010), and histological reports (Eidelberg and Galaburda 1982). In fact, the reported cerebral asymmetries in the brain are mainly related to its functional activity. For instance, language functions are historically known to be left-lateralized, while those involved in spatial orientation and emotional control are predominantly associated with the right hemisphere (Rimol et al. 2006; Toga and Thompson 2003). In our study, since we recruited only right-handed subjects, we could expect possible inter-hemispheric differences between the groups of nuclei involved in motor control (i.e. the VLV) because of the largely known motor-related lateralization of the brain (Gut et al. 2007). To the best of our knowledge, there is no evidence of strong structural left–right asymmetries in the spatial organization of the thalamic nuclei, and our findings are in line with that. Another reason for the absence of hemispheric asymmetries can be attributed to the low spatial resolution of the DWI acquisition in comparison to the small size of the thalamus. This represents a limitation of our study that is shared with all the previous published research on the same topic.

The findings from the core data (Dataset 1) were also observed in the analyses of the additional two datasets, proving the reproducibility of the outcome over different diffusion sequences and different scanner machines. Moreover, in a scan–rescan scenario, with very high Dice values considering the relative small size of the clusters, we show strong reproducibility of the results over different time points, and therefore, we reinforce the validation of our findings. The reproducibility of the test-retest analysis is also proven by both centroids and borders distances, which are always smaller than the original spatial resolution of the used diffusion data.

We also performed a long-connection tractography-based analysis to further evaluate the robustness of our clustering algorithm and the ability to identify appropriate anatomical pathways described in the literature (Jones, 1985). We observed a high frequency of success (FS) for the expected overlaps, 92.4% in average, which further supports the anatomical accuracy of the spatial distribution of the segmented clusters. We want to emphasize the fact that such evaluation has no intent of comparing fiber-tracking-based clustering with our local diffusion property-based clustering. Instead, to provide additional anatomical value to our results, we tested the hypothesis that the main thalamo-cortical fibers characteristic of a nucleus should pass through it.

Our clustering method, which is based on local diffusion properties, is a robust tool for thalamic nuclei segmentation that closely matches histological atlases. We showed that our method outperforms recent state-of-the-art methods based on local diffusion properties, or more precisely, the angular difference (AD), in terms of reproducibility and parcellation matching closely with the known anatomical architecture of the thalamus. Moreover, the AD-based segmentation outcome presented less smooth cluster boundaries for diffusion data acquired with *b* values of 2000 s/mm^2^. We assume this is due to the limitations of the tensor modeling that fails to represent properly the additional diffusion information, presumably coming from the intra-voxel compartments (Baumann et al. 2012) that such data provide.

Our results differ from cortical connectivity-based approaches results (Behrens et al. 2003; O’Muircheartaigh et al. 2011), which generally found overlapping connections to multiple cortical areas as well as great inter-subject variability. Several factors may have contributed to this result. First, the cortical target ROIs used for tractography were large and characterized by fuzzy borders, which favored the existence of multiple cortical connections from each connectivity-defined thalamic region. Second, the diffusion tractography is sensitive to major pathways, and therefore, smaller pathways, especially if crossing other tracts, are not always detected. Third, the thalamus is a very complex structure, characterized by different cell types and specific cortical connections (matrix and core neurons; Jones 2001) which can bias the results of long-connection fiber tractography. It should also be considered that the thalamus segmentation by fiber-tracking does not necessarily correspond to an anatomical subdivision of the thalamus (Behrens et al. 2003; Deoni et al. 2005; O’Muircheartaigh et al. 2011; Traynor et al. 2011). This is particularly true for nuclei connected to the sensorimotor cortex, whereas good correspondence was found for the pulvinar, the thalamic nucleus mainly projecting to the occipital cortex (Shipp 2003). This scenario has also been replicated in functional-based connectivity studies (Hale et al. 2015). They revealed distinct features of thalamo-cortical connectivity (Zhang et al. 2010) when compared to structural-based ones, showing that these two methodologies provide complementary information. As with tractography-based approaches, they share the same problems of overlapping of connectivity and inter-subject variability.

With the aim of providing a tool of a potential interest in everyday clinical practice, we estimated the ODFs using a DWI sequence typically acquired in a clinical setting. As a drawback, we share the limitation of all the other published studies so far that are related to the low spatial resolution of the DWI sequences. With a voxel size of approximately 2 x 2 x 2 mm^3^, we were not be able to distinguish smaller nuclei or nuclear groups. Complementary techniques should be additionally considered to drive the segmentation towards smaller anatomical subdivisions. These include high angular acquisition schemes, such as diffusion spectrum imaging (DSI), which has been demonstrated to better characterize crossing fibers (Wedeen et al. 2008), and/or the use of high-field MRI scanner. For instance, it has been shown that susceptibility-weighted imaging acquired at 7 T (Abosch et al. 2010), is able to provide complementary information to those extracted from DWI about thalamic microstructure, which can help in delineating the different groups of thalamic nuclei. Future studies may also include the acquisition of diffusion images at higher *b* values (>2000 s/mm^2^), and/or the use of scanner with higher gradient systems.

With our approach, we were able to show robust and anatomically consistent segmentation of the main groups of thalamic nuclei. Thus, our framework can be of potential use in many clinical applications. We would like to emphasize that unlike the cortical connectivity-based algorithms, relying on local diffusion properties may be an important asset when studying patients that have moderate or severe lesions in WM or GM (such as tumors, stroke or vascular lesions), for whom long-distance fiber-tracking may fail. Other examples of possible applications can be related to movement disorders. Our recent findings (Battistella et al. 2013) in young asymptomatic *FMR1* premutation carriers at risk of developing a late-onset movement disorder called fragile X tremor–ataxia Syndrome (FXTAS), encourage further evaluation of the motor-control pathway and in particular, the thalamic ventral intermediate nucleus (Vim) that is part of this network (included in the VLV group in this study). Similarly, the VLV delineation is of potential interest to clinical studies and treatment planning for other movement-related disorders, such as essential tremor, where the central element is again the Vim (Ohye et al. 2012). The automatic delineation of all seven groups of nuclei also represent a useful tool for studies related to brain development (Jones 1997) or to better interpret functional studies.

## Conclusion

We propose a novel automated framework for segmenting the thalamic subparts, which explores the orientation distribution functions represented in spherical harmonics basis from diffusion MR images at 3 T. The ability to fully characterize the crossing fibers, in addition to a data-driven initialization of the clustering algorithm, provides a robust, reproducible and an accurate segmentation of seven groups of thalamic nuclei that outperforms the current state-of-art based on local diffusion properties. Each segmented nuclei group has a characteristic spatial distribution, which closely matches histological atlases, and identifies a major cortico-thalamic pathway.

## Electronic supplementary material

Below is the link to the electronic supplementary material.

Supplementary material 1 (DOCX 2749 kb)
