# Supplementary material for: Robust thalamic nuclei segmentation method based on local diffusion magnetic resonance properties
Source: Brain Struct Funct. 2016 Nov 25;222(5):2203–16. doi: 10.1007/s00429-016-1336-4 (PMC5504280; doi:10.1007/s00429-016-1336-4)

**Supplementary material:**

**Figure SM1.** Single-subject comparison with the axial slice D 4.5 of Morel’s atlas

**
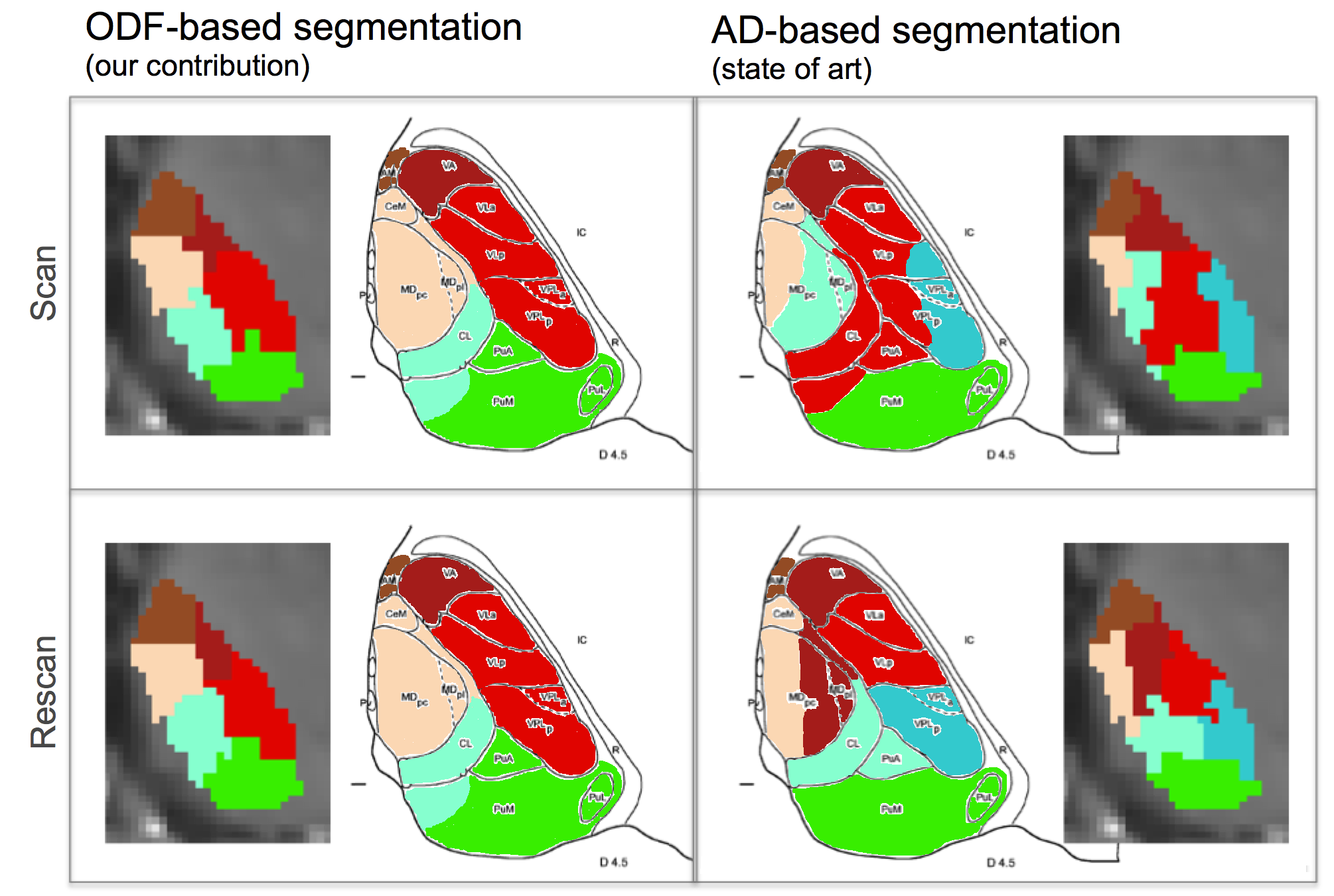
**

**Figure SM2.** Single-subject comparison with the axial slice D 10.8 of Morel’s atlas

**
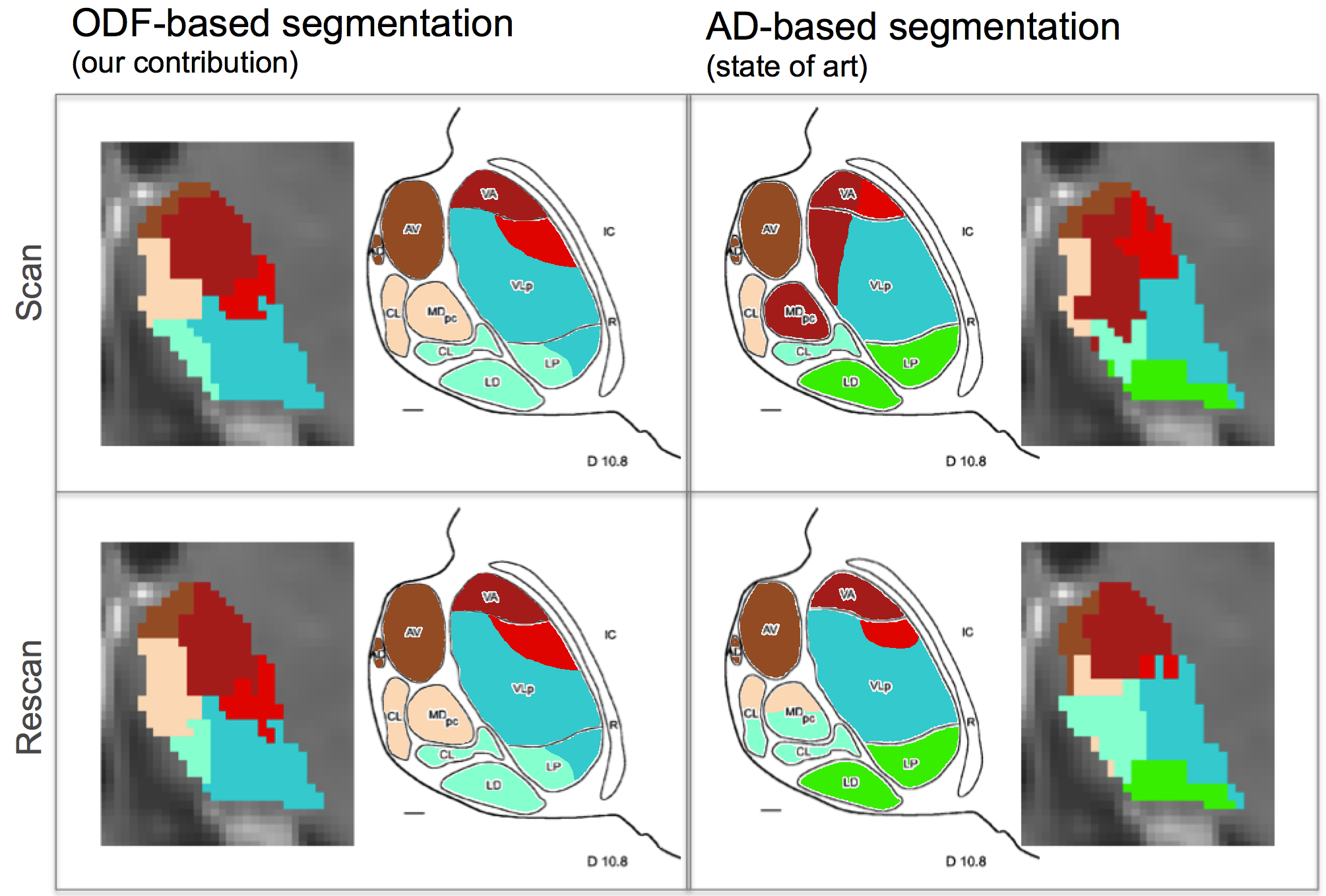
**

**Figure SM3.** Reconstruction of thalamic long connections. 3D views of the reconstructed fiber tracts (in white) for the Medio-Dorsal group (panel A) and the Pulvinar (panel B). Probabilistic tracts were reconstructed using the whole thalamus mask and the following seed regions (in yellow): left middle frontal sulcus and amygdala for the MD group, and left inferior angular gyrus and left calcarine sulcus for the Pulvinar.


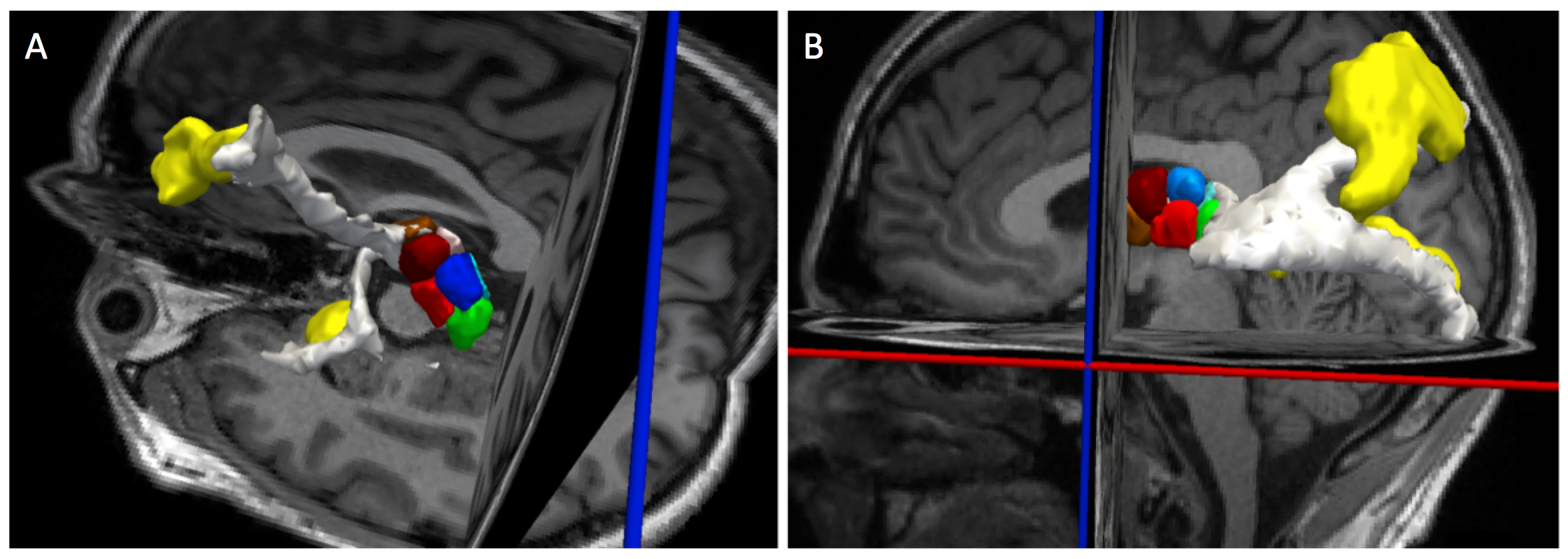

Supplement: Supplementary file 1 — Supplementary material 1 (DOCX 2749 kb) [file 429_2016_1336_MOESM1_ESM.docx]
